# The efficacy of freehand, pilot drilled and fully guided implant surgery in partially edentulous patients: A randomized control trial

**DOI:** 10.1371/journal.pone.0341894

**Published:** 2026-01-27

**Authors:** Abdulkhaliq Ali F. Alshadidi, Lujain Ibrahim N. Aldosari, Abdullah Hasan A. Alshehri, Rayan Ibrahim H. Binduhayym, Rajamanoj Kondaveeti, Vishwanath Gurumurthy, Sunil Kumar Vaddamanu

**Affiliations:** 1 Department of Allied Dental Health Sciences, College of Applied Medical Sciences, King Khalid University, Abha, Saudi Arabia; 2 Department of Prosthodontics, College of Dentistry, King Khalid University, Abha, Saudi Arabia; 3 Correctional Unit Dentist State of Texas, Affiliated With Texas Tech University of Health Sciences, Abilene, Texas, United States Of America; LSU Health Shreveport, UNITED STATES OF AMERICA

## Abstract

**Background:**

Partial edentulism poses challenges to oral function, aesthetics, and quality of life. Implant placement techniques—freehand, pilot-drilled, and fully guided—differ in accuracy, surgical time, and outcomes. In this study, only one predefined index implant per patient was analyzed to avoid confounding from multi-implant cases, and template fabrication for the pilot-drilled group was performed using diagnostic wax-up and thermoplastic material. This study evaluated these techniques in partially edentulous patients.

**Methods:**

Ninety patients were randomly assigned to three groups: freehand (n = 30), pilot- drilled (n = 30), and fully guided (n = 30). Surgery duration, implant placement accuracy, post- operative complications, early implant failure rates, and patient satisfaction were measured. Accuracy was assessed using standardized CBCT imaging at 12 months, and satisfaction was evaluated via a validated questionnaire six months after prosthetic loading.

**Results:**

The fully guided technique demonstrated superior accuracy (p < 0.001), shorter surgical times (45 minutes vs. 60 and 75 minutes, p < 0.01), fewer complications (5% vs. 15% and 20%, p < 0.05), and higher satisfaction (9.2/10, p < 0.01). Early implant failure, defined at the implant level, occurred in 4/30 implants (13.3%) in the freehand group, 0/30 in the pilot-drilled group, and 2/30 in the fully guided group (p < 0.05).

**Conclusion:**

Fully guided implant surgery outperformed other techniques in accuracy, efficiency, and patient satisfaction. These findings support fully guided, prosthetically driven workflows as a preferred option for partially edentulous patients, particularly in cases requiring high precision.

## Introduction

Partial edentulism refers to the condition where one or more natural teeth are absent in an otherwise intact dental arch. This condition can significantly impact a patient’s ability to chew, speak, and maintain proper oral hygiene, leading to a decrease in quality of life. The psychological impact is equally substantial, as missing teeth can alter a patient’s self-esteem and social interactions. With advancements in dentistry, dental implants have emerged as a reliable solution for restoring missing teeth, allowing for improved function and aesthetics. However, the success of these implants is highly contingent upon the surgical techniques employed during the placement. [1–3]. The surgical technique used plays a major role in determining three-dimensional implant positioning, emergence profile, peri-implant bone stability, and overall treatment longevity.

Accurate implant placement is critical because even small deviations in angulation, apical or coronal position, or depth can adversely affect biomechanical loading, esthetic outcomes, and soft-tissue behavior. Malposition implants may lead to compromised gingival contours, inadequate interproximal papillae, impaired occlusal loading, or peri-implant bone loss [4–7]. The precision of implant placement is influenced by anatomical conditions, operator expertise, and the inherent capability of the chosen surgical protocol. Consequently, precision and reproducibility have become central elements when evaluating implant placement methods.

In addition to precision, esthetics has become a focal point in implant dentistry, especially in the anterior maxilla, where soft-tissue morphology and prosthetic harmony are highly visible. Esthetic failures often stem from inaccurate implant angulation or incorrect three-dimensional positioning, underscoring the importance of techniques that can predictably support esthetic outcomes [2,5].

Anatomical constraints such as limited bone volume, ridge resorption patterns, proximity to vital structures, and anatomical curvature introduce significant challenges during implant planning. Cone-beam computed tomography (CBCT) has therefore become essential for preoperative evaluation, enabling clinicians to assess bone quality, morphology, and spatial relationships [8–10]. Furthermore, in modern digital workflows, prosthetically driven planning serves as a critical bridge between the surgeon and prosthodontist, ensuring that implant positioning is determined primarily by the ideal restorative outcome and then anatomically validated. This coordinated approach enhances esthetic predictability, functional loading, and long-term biomechanical success.

Several surgical approaches have been developed to facilitate accurate implant placement. Freehand placement relies heavily on the clinician’s visual and tactile judgment, which may introduce variability, especially in complex anatomical situations [5–7,11]. The pilot-drilled (partially guided) technique uses a simple, tooth-supported template fabricated from diagnostic wax-ups and thermoplastic or vacuum-formed materials to guide only the initial osteotomy. This template helps establish the correct entry point and angulation of the pilot drill, after which the remaining osteotomy sequence is completed freehand. While this method provides better initial directional control than fully freehand placement, it still allows operator-dependent deviations during subsequent drilling steps, which may influence final implant position [12–16]. Unlike previous studies that compared freehand and partially guided approaches using non-standardized templates or inconsistent surgical sequences, the current trial implemented a rigorously standardized protocol for all three techniques—freehand, pilot-drilled, and fully guided—including uniform guide fabrication, CBCT-based measurements, and identical implant systems, thereby eliminating methodological variability seen in earlier research.

Fully guided surgery, supported by CBCT-driven digital planning and stereolithographic surgical guides, offers controlled osteotomy preparation with predefined angulation, depth, and mesiodistal or buccolingual positioning, and has been associated with improved accuracy, reduced complications, and enhanced patient satisfaction [8,17–22].

Most published studies evaluating fully guided implant surgery—across both anterior and posterior regions—have consistently demonstrated superior accuracy compared with freehand placement, reporting mean coronal deviations between 0.6–1.2 mm, apical deviations between 0.8–1.5 mm, and angular deviations of 2–4° [5,7,8,23–25]. In contrast, evidence on partially guided (pilot-drill) workflows remains limited and methodologically inconsistent, with reported angular deviations ranging widely from 3° to more than 8°, depending on guide design, support type, and operator experience [13–15]. This variability reflects substantial heterogeneity in protocols, measurement techniques, and clinical settings, making cross-study comparisons challenging and highlighting the need for standardized randomized trials.

The aim of this randomized controlled trial was to systematically compare the clinical accuracy, surgical efficiency, postoperative recovery, implant stability, and patient-reported outcomes associated with freehand, pilot-drilled, and fully guided implant placement techniques in partially edentulous patients.

We hypothesized that fully guided implant surgery would demonstrate significantly greater placement accuracy, fewer complications, reduced postoperative discomfort, and higher patient satisfaction compared with pilot-drilled and freehand approaches, owing to its enhanced surgical control and prosthetically driven planning workflow.

## Materials and methods

### Study design

Partially edentulous patients (those missing one or more teeth in an arch but with remaining natural teeth) would be recruited for the study. This study received ethical approval from the Research Ethics Committee at King Khalid University’s under Approval no. ECM#2024–3195. Additionally, it was registered with ClinicalTrials.gov, identifier no: NCT06764784. The authors confirm that all ongoing and related trials for this drug/intervention are registered. All patients were of Middle Eastern/Arab ethnicity, and all implant surgeries were performed in standardized private clinical settings located in Abha and Sabya, Saudi Arabia, following identical infection-control and operative protocols.

A total of 90 partially edentulous patients were randomly assigned from 6^th^ January 2024 to 3^rd^ February 2024 to one of three groups: freehand (n=30), pilot-drilled (n=30), and fully guided (n=30) implant placement. Patient screening and randomization were completed between 6 January 2024 and 3 February 2024. All implant surgeries were performed between 10 January 2024 and 20 February 2024, and patients were followed for 12 months after implant placement. Clinical follow-up therefore extended from January 2024 to February 2025. Implant stability (ISQ) and radiographic outcomes were recorded at baseline (implant insertion), 3, 6, and 12 months, while patient-reported satisfaction was evaluated at 6 months postoperatively.

The sample size was calculated a priori using G*Power 3.1.10 (Heinrich-Heine-Universität Düsseldorf, Germany). The primary outcome for power estimation was implant placement accuracy (combined coronal, apical, depth, and angular deviation values). Based on effect sizes reported in previous randomized trials comparing guided vs. freehand placement, a conservative medium effect size of Cohen’s f = 0.30 (η² ≈ 0.08) was assumed. For a one-way ANOVA with three parallel groups, α = 0.05 and power (1−β) = 0.80, the minimum required sample was 81 patients. To compensate for up to 10% attrition, dropouts, or protocol deviations, the sample was increased to 90 participants (n = 30 per group), ensuring adequate power for both primary and secondary outcomes.

Although the sample size was modest, it was adequately powered for the primary and secondary outcomes. Additionally, block randomization and surgeon calibration were implemented to minimize variability, reduce confounding, and enhance internal validity despite the relatively small cohort.

To minimize operator-related confounding, randomization was conducted using a computer-generated block randomization sequence (block size = 6), stratified by jaw (maxilla vs. mandible) and implant site (anterior vs. posterior) to ensure balanced anatomical distributions across the three groups. All surgeries were performed by two implantologists with comparable experience (each >10 years of clinical practice and >500 implant placements). Prior to initiating the trial, both surgeons underwent a structured calibration protocol consisting of: (1) harmonization of the osteotomy sequence for all three techniques; (2) a joint training session on five non-study pilot cases; and (3) CBCT-based inter-operator reproducibility assessment. Inter-operator reliability was excellent, with an intraclass correlation coefficient (ICC) of 0.92 for angulation and 0.89 for depth deviation, confirming high procedural consistency.

Although some patients presented with more than one missing tooth, only one implant per patient (the predefined index implant) was included in the study analyses to avoid confounding related to surgical duration, postoperative discomfort, or stability values. Clinically, a small subset of patients required two implants; however, these additional implants were not included in the analytic dataset. The distribution of clinically placed implants per patient is summarized in Table 1. Because randomization was stratified by jaw (maxilla vs. mandible) and implant site (anterior vs. posterior), the distribution of missing spaces across the three groups was comparable. This ensured that differences in implant position (canine, premolar, or molar regions) did not confound outcome comparisons.

**Table 1:** Distribution of clinically placed implants per patient.

Study Group	Total Patients (n)	Patients with 1 implant placed (clinical)	Patients with 2 implants placed (clinical)	Total implants actually placed (clinical)	Implants included in analysis (index implant)
**Freehand**	30	24	6	36	30
**Pilot-drilled**	30	25	5	35	30
**Fully guided**	30	26	4	34	30

**Fig 1:**
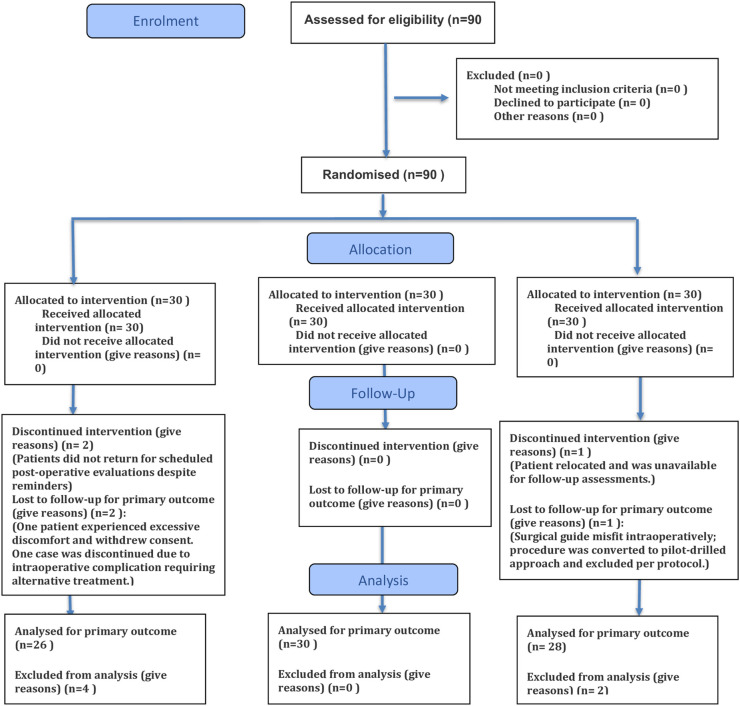
CONSORT 2025 flow diagram.

Key parameters measured included surgery duration, implant placement accuracy, post- operative complications, early implant failure rates, and patient satisfaction. Accuracy was assessed with post-operative radiological imaging, and patient satisfaction was measured using a validated questionnaire at six months post-surgery. Fig 1. [26]

#### Inclusion criteria.

- Adults aged 18 years or older.
- Patients presenting with partial edentulism.
- Sufficient bone volume and quality for implant placement.
- No systemic contraindications for surgery.

#### Exclusion criteria.

- Patients with uncontrolled systemic diseases.
- Smokers or those with a history of non-compliance with post-operative care.
- Patients requiring additional bone grafting or sinus lifts.

A single implant system was used for all patients: Straumann® Bone Level Tapered implants (Institut Straumann AG, Basel, Switzerland; titanium grade 4, sandblasted and acid-etched surface) with diameters of 3.5–4.3 mm and lengths of 8–12 mm, selected according to the available bone as determined on CBCT. All fixtures featured an internal connection and platform-switching design to standardize prosthetic and biological behavior.

Patients would be randomly assigned to one of three groups

1. Freehand Implant Surgery (FHIS)
2. Pilot Drilled Implant Surgery (PDIS)
3. Fully Guided Implant Surgery (FGIS)

#### Pre-operative assessment.

- Comprehensive dental and medical history.
- Clinical examination.
- Radiographic assessment was performed using a cone-beam CT unit (Planmeca ProMax® 3D Mid, Planmeca Oy, Helsinki, Finland) with the following parameters: 90 kVp, 10 mA, voxel size 0.2 mm, and exposure time 12–15 s. A jaw-specific field of view (FOV 8 × 8 cm) was used to minimize radiation while encompassing the planned implant site. DICOM data were exported and analyzed using the manufacturer’s imaging software (Romexis®, Planmeca Oy) for measurements of bone height and width and to identify critical anatomical structures.
- Impressions for study models to plan the implant positions.

The edentulous space in each patient was evaluated clinically and radiographically to determine suitability for implant placement. Clinical criteria included verification of mesiodistal space, occlusal clearance, keratinized tissue width, and neighboring tooth angulation. Radiographically, CBCT was used to assess available bone height, width, and proximity to anatomical structures (mental foramen, maxillary sinus, adjacent roots). Based on these criteria, the most favorable missing space (canine, premolar, or molar region) was selected as the planned implant site. To avoid selection bias, stratified block randomization ensured that anterior and posterior sites were evenly distributed across the three groups.

### Surgical protocols

All surgeries were performed by two calibrated implant surgeons (Dr. S.K. and Dr. A.K.), each with over 10 years of implant experience. Accuracy measurements and radiographic analyses were performed by an independent examiner (Dr. V.G.), who was not involved in the surgical procedures.

#### Freehand Implant Surgery (FHIS).

For the freehand implant surgery (FHIS) group, all procedures were performed under local anesthesia using a standardized flap approach. A mid-crestal incision with releasing incisions, when required, was made to expose the alveolar ridge. Osteotomy preparation was carried out freehand using the manufacturer’s sequential drill protocol under copious sterile saline irrigation, without the use of any surgical guide. Drill angulation and depth were controlled visually using adjacent teeth and anatomical landmarks. Implants were inserted with a target insertion torque of ≥35 Ncm, and primary stability was verified clinically and with ISQ measurements. After placement, cover screws were installed, and the flaps were repositioned and sutured with 4-0 resorbable sutures. All patients received standardized postoperative care (analgesics, 0.12% chlorhexidine mouth rinse, and written hygiene instructions).

#### Pilot Drilled Implant Surgery (PDIS).

In the pilot-drilled implant surgery (PDIS) group, a tooth-supported pilot template was fabricated from a diagnostic wax-up using a vacuum-formed thermoplastic sheet. The template guided only the 2.0-mm pilot drill to establish the planned entry point and angulation. After template-guided pilot drilling, the template was removed and the osteotomy was completed freehand using the manufacturer’s sequential drills under copious saline irrigation. No fluoroscopy was used in this study; instead, pilot drill positioning was verified clinically and, when necessary, with a periapical radiograph taken using a paralleling technique. Implants were then inserted to the planned depth with a target insertion torque ≥35 Ncm. Cover screws were placed, and flaps were sutured as in the FHIS group. Postoperative medication and instructions were identical across all groups

#### Fully Guided Implant Surgery (FGIS).

For the fully guided implant surgery (FGIS) group, preoperative planning was performed on CBCT data sets imported into dedicated implant planning software (coDiagnostiX®, Dental Wings, Chemnitz, Germany). A digital diagnostic wax-up was merged with the CBCT to enable prosthetically driven, three-dimensional planning of implant position, angulation, and depth. Tooth-supported stereolithographic surgical guides were then designed and printed in biocompatible resin with metal sleeves (layer thickness 50–100 µm) using a desktop 3D printer. During surgery, the guide was seated and verified for complete seating and stability on the remaining dentition. Accurate guide seating was confirmed by visual inspection of all tooth-supported rest points, tactile verification using finger pressure, and ensuring that no rocking or micro-movement occurred. In addition, unobstructed seating of the metal sleeves over the planned drill trajectories was checked before initiating osteotomy. All osteotomy steps, including drilling sequence and depth control, were performed through the guide sleeves using the compatible guided drill kit provided by the implant manufacturer. Implants were inserted through the guide in the planned orientation. After removal of the guide, cover screws were placed, and the soft tissues were sutured as in the other groups.

Following implant placement, all implants were treated with a conventional delayed loading protocol. Cover screws were placed, and the implants were left submerged for approximately 3 months. At 3 months, a second-stage surgery was performed to place healing abutments, followed by definitive prosthetic rehabilitation between 4 and 6 months after surgery. Thus, all implants were in functional loading for at least 6 months by the 12-month follow-up visit. Patient-reported satisfaction was assessed 6 months after prosthetic loading, while ISQ and radiographic measurements were repeated at 3, 6, and 12 months.

#### Outcome measures.

The efficacy of each technique can be assessed through various outcomes Table 2.

**Table 2:** Outcome measures of each technique.

Outcome Category	Measure
**Surgical Parameters**	Duration of surgery (time taken for implant placement)
	Number of successful vs. failed implant placements
	Intraoperative complications (e.g., bleeding, perforation)
**Post-operative Outcomes**	Pain scores (Visual Analog Scale)
	Swelling and Discomfort post- surgery
	Healing index and soft tissue response
	Radiographic assessment of implant position and integration (CBCT at 3,6,12 months)
**Implant stability and Osseointegration**	Resonance frequency analysis at 3,6,12 months
	Radiographic assessment of peri-implant bone changes and osseointegration over time.
**Patient satisfaction**	Surveys on aesthetics, comfort and satisfaction with prosthesis
**12-month survival and complications**	Implant survival at 12 months; incidence of biological and mechanical complications;
	Marginal bone level change (mm) at 12 months

Peri-implant marginal bone levels were assessed on standardized periapical radiographs taken with a long-cone paralleling technique at baseline (prosthesis delivery), 6 months, and 12 months. Mesial and distal bone levels were measured from the implant–abutment junction to the first bone-to-implant contact using calibrated digital software, and changes over time were expressed in millimeters. This assessment reflected vertical marginal bone remodeling rather than horizontal ridge width changes.

### Statistical analysis

Normality was assessed using Shapiro–Wilk tests and QQ plots. One-way ANOVA was used to compare the three techniques on cross-sectional continuous outcomes: surgery duration (minutes), implant placement accuracy (entry point, apex, depth deviation in mm; angulation in degrees), and patient satisfaction (VAS 0–10 at 6 months), followed by Tukey post hoc tests. Repeated-measures implant stability (ISQ) and time-to-event early failure data were analyzed using the longitudinal and survival methods described hereafter. Categorical outcomes (complications and early implant failure) were analyzed using Fisher’s exact test to assess whether their distributions differed significantly across the three surgical groups. Non-parametric Kruskal–Wallis exact tests (Monte Carlo, 10 000 samples) were performed as sensitivity analyses and confirmed all parametric findings. All tests were two-tailed, α = 0.05, using SPSS version 23 (IBM Corp., Armonk, NY).

#### Subgroup analysis by implant position.

Implants were placed in either canine or molar regions depending on the edentulous site. To evaluate whether implant position (canine vs. molar) influenced the outcomes across surgical techniques, a subgroup analysis was planned a priori and stratified by tooth position. Specifically, for each outcome variable (surgery duration, accuracy deviations in entry point, apex, depth, and angulation; postoperative complications; early failure rates; and patient satisfaction), data were separated into canine (n = 42 implants) and molar (n = 48 implants) subgroups across all 90 patients. A two-way ANOVA was conducted with surgical technique (freehand, pilot-drilled, fully guided) as the primary factor and implant position (canine vs. molar) as a secondary factor, including an interaction term. No significant interaction effects were detected (all p > 0.10), indicating that the effect of surgical technique on outcomes did not vary with the implant position. Main effects of position were also non-significant for all variables (p > 0.05) except surgery duration, where molar sites required slightly longer times on average (+8.3 minutes, p = 0.04) due to anatomical access, but this did not alter group comparisons. Therefore, results are reported for the overall sample, with subgroup consistency supporting the generalizability of findings across anterior and posterior regions.

#### Longitudinal and survival analysis.

Implant stability was assessed using resonance frequency analysis (RFA) and expressed as Implant Stability Quotient (ISQ) values at baseline (implant insertion) and at 3, 6, and 12 months postoperatively. Early implant failure was defined at the implant level as loss of osseointegration, clinical mobility, removal of the implant, or inability to prosthetically restore the implant within 12 months; it was recorded as a time-to-event outcome (months to failure or censoring at 12 months).

Longitudinal ISQ data were analyzed using linear mixed-effects models (LMM) with fixed effects for surgical technique, time point, and their interaction, and a random intercept per patient. The covariance structure for the repeated measures was selected as the one yielding the lowest Akaike Information Criterion (AIC); this was a first-order autoregressive structure with heterogeneous variances (AR(1)-heterogeneous).

The AR(1)-heterogeneous covariance structure was specified for the within-patient residual errors, conditional on the random intercept. Thus, correlation between repeated ISQ measurements was modeled through two components: a random intercept capturing between-patient heterogeneity, and an AR(1)-heterogeneous residual structure accounting for time-dependent within-patient correlation and unequal residual variances across follow-up visits. An explicit residual error term was therefore included in the model.

Pairwise comparisons were Bonferroni-adjusted. Survival analysis of early implant failure was performed using Kaplan–Meier curves with log-rank tests and Cox proportional hazards regression (hazard ratios with 95% CI). The proportional hazards assumption was assessed using Schoenfeld residuals. Accuracy assessment included quantifying coronal, apical, depth, and angular deviations using standardized CBCT planning software, with the implant platform center and apex serving as reference points for deviation calculations. Postoperative verification of implant position and accuracy was performed using a limited-FOV CBCT scan acquired with the same device and parameters as the preoperative scan. Pre- and postoperative CBCT datasets were superimposed in the planning software to quantify coronal, apical, depth, and angular deviations between planned and inserted implant positions.

## Results

Of the 90 randomized patients, 88 completed the 12-month follow-up. Two patients (one in the freehand group and one in the fully guided group) were lost to follow-up due to relocation and were excluded from the final analysis. The sample size had been inflated by 10% during planning to accommodate potential attrition; therefore, this dropout did not compromise statistical power or the validity of group comparisons. Baseline characteristics of the two dropouts did not differ meaningfully from those who completed the study, and no pattern of selective attrition was observed.

### Demographics

Although some patients received more than one implant clinically, only one predefined index implant per patient was included for analysis to ensure comparability across groups and avoid confounding related to variable surgical burden. Table 3

**Table 3:** Baseline demographic and clinical characteristics.

Variable	Freehand (n = 30)	Pilot-drilled (n = 30)	Fully guided (n = 30)	p-value
**Age (years, mean ± SD)**	54.6 ± 10.2	55.1 ± 11.4	55.3 ± 9.8	0.74
**Gender (Male/Female)**	15/15	14/16	16/14	0.88
**Jaw treated (Maxilla/Mandible)**	17/13	16/14	18/12	0.81
**Site (Anterior/Posterior)**	12/18	13/17	14/16	0.69
**Patients with 1 implant placed clinically**	24	25	26	–
**Patients with 2 implants placed clinically**	6	5	4	–
**Implants included in analysis**	30	30	30	–

**Fig 2:**
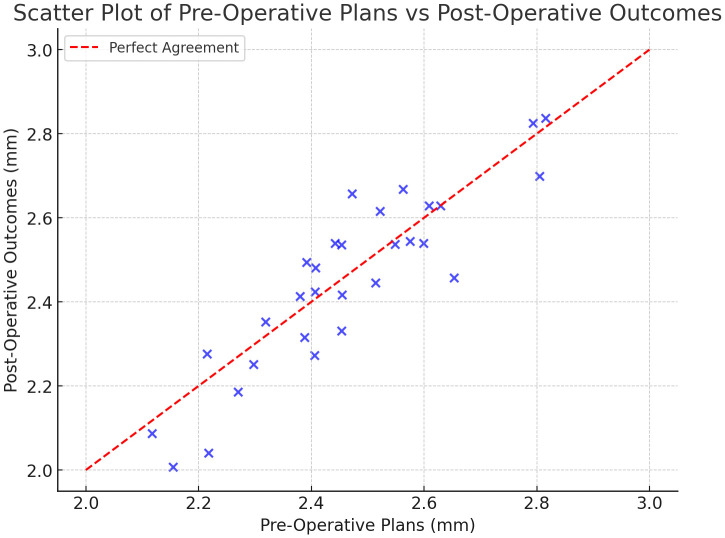
This shows the relationship between pre-operative plans and post-operative outcomes. The dashed red line represents perfect agreement, with points clustering around it indicating accuracy.

Baseline equivalence across the three randomized groups was evaluated to confirm that randomization successfully balanced potential confounders. Age, gender distribution, jaw involved (maxilla vs. mandible), implant site (anterior vs. posterior), and baseline bone density were compared across groups using one-way ANOVA for continuous variables and Fisher’s exact test for categorical variables. No statistically significant differences were detected for age (p = 0.74), gender (p = 0.88), jaw distribution (p = 0.81), implant site (p = 0.69), or baseline bone density (p = 0.63).These findings indicate that the randomization procedure provides a reasonable balance of all measured demographic and anatomical confounders across the three comparison groups. The fully guided group demonstrated significantly higher accuracy in implant placement compared to both the pilot-drilled and freehand groups (p < 0.001). Additionally, surgery duration was shortest in the fully guided group, with a mean time of 45 minutes compared to 60 minutes for the pilot-drilled and 75 minutes for the freehand methods (p < 0.01). Post-operative complications occurred in 5% of the fully guided group, while complications were noted in 15% and 20% of the pilot-drilled and freehand groups, respectively (p < 0.05) Fig 2.

**Fig 3:**
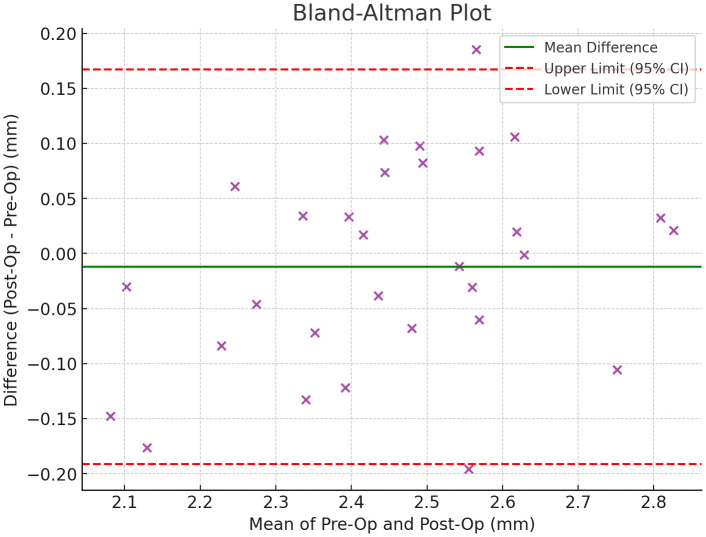
This highlights the agreement between pre-operative and post-operative values. The green line represents the mean difference, while the dashed red lines mark the 95% confidence limits for differences.

Early implant failure, defined at the implant level, occurred in 4/30 implants (13.3%) in the freehand group, 0/30 implants (0%) in the pilot-drilled group, and 2/30 implants (6.7%) in the fully guided group (p < 0.05). Patient satisfaction ratings were highest in the fully guided group at 9.2/10, compared to 8.5/10 and 7.2/10 in the pilot-drilled and freehand groups (p < 0.01) (Tables 4–, Fig 3).

**Table 4:** Outcome measures of each technique.

Parameter	Freehand	Pilot-Drilled	Fully Guided
**Surgery Duration (min)**	Avg 75 (Range 50–75)	Avg 60 (Range 25–60)	Avg 45 (Range 30–55)
**Successful Implant Placements**	28/30 (93.3%)	30/30 (100%)	29/30 (96.7%)
**Intraoperative Complications**	3 (10%)	2 (6.7%)	1 (3.3%)

**Table 5:** Postoperative recovery.

Parameter	Freehand	Pilot-Drilled	Fully Guided
**Pain Level (VAS)**	Mean 5.2	Mean 4.5	Mean 3.8
**Postoperative Swelling**	3.5 cm (20%)	2.8 cm (15%)	2.2 cm (5%)

**Table 6:** Implant Stability (RFA) & Osseointegration.

Parameter	Freehand	Pilot-Drilled	Fully Guided
**ISQ at 3 months**	65	70	75
**ISQ at 6 months**	68	72	78
**ISQ at 12 months**	70	74	80
**Bone Density (HU)**	350 ± 60 → 300 ± 70 at 12 months	360 ± 50 → 310 ± 60	365 ± 55 → maintained higher stability
**Peri-implant Bone Height Loss (12 months)**	2.0 ± 0.5 mm	1.5 ± 0.6 mm	0.5 ± 0.3 mm

**Table 7:** Patient satisfaction & long-term success.

Parameter	Freehand	Pilot-Drilled	Fully Guided
**Overall Satisfaction (0–10)**	7.2	8.5	9.2
**Aesthetic Satisfaction**	7.0	7.8	8.5
**Implant Survival at 12 Months**	26/30 (86.7%)	30/30 (100%)	28/30 (93.3%)
**Incidence of Biological/Mechanical Complications**	5 (16.7%)	2 (6.7%)	1 (3.3%)

### Longitudinal Implant Stability (ISQ)

Linear mixed-effects modelling showed significant main effects of surgical technique (F(2,87)=18.42, p < 0.001) and time (F(3,261)=142.6, p < 0.001), and a significant surgical technique × time interaction (F(6,261)=4.31, p = 0.002). Because this interaction was significant, the main effects of technique and time cannot be interpreted independently. The interaction indicates that the pattern of ISQ change over the 12-month follow-up differed among the three surgical groups. A significant interaction indicates that the pattern of ISQ change over the 12-month follow-up differed among the three surgical groups. These analyses were then followed with basic contrasts to compare the longitudinal trends within each time interval among the three study groups differ, or any two groups..

Post-hoc contrasts demonstrated that the fully guided group had significantly higher ISQ values than both the pilot-drilled and freehand groups at every follow-up interval (Bonferroni-adjusted p < 0.05). The largest difference occurred at the 3-month visit (fully guided: 78.4 ± 4.1 vs. pilot-drilled: 74.8 ± 4.9 and freehand: 72.1 ± 5.3). Additionally, the fully guided group exhibited the steepest longitudinal increase in implant stability, with a + 19-unit ISQ gain from baseline to 12 months, indicating a more favorable osseointegration trajectory compared with the other techniques. (Table 8).

**Table 8:** Implant Stability Quotient (ISQ) over time by surgical technique (Mean ± SD); overall surgical technique x time interaction p-value from linear mixed effects model.

Time Point	Freehand (n = 30)	Pilot-drilled (n = 30)	Fully guided (n = 30)
Baseline	62.1 ± 4.8	63.4 ± 4.5	64.8 ± 4.2
3 months	72.1 ± 5.3	74.8 ± 4.9	78.4 ± 4.1
6 months	75.6 ± 5.1	77.9 ± 4.7	81.2 ± 3.8
12 months	79.2 ± 4.9	81.7 ± 4.4	83.8 ± 3.6

The surgical technique × time interaction was evaluated as a single global effect across all four time points using a linear mixed-effects model (p = 0.002). Time-specific between-group differences were assessed using Bonferroni-adjusted post-hoc contrasts and are described in the text.

Planned contrasts revealed that the largest between-group divergence in ISQ trajectories occurred during the early healing phase (baseline to 3 months). During this interval, the fully guided group showed a significantly greater increase in ISQ compared with both the pilot-drilled and freehand groups (Bonferroni-adjusted p < 0.01). Between 3 and 6 months, ISQ values continued to increase in all groups, with smaller but still significant differences favoring the fully guided group over freehand placement (p < 0.05), while differences between fully guided and pilot-drilled techniques were attenuated. No statistically significant differences in ISQ change were observed between 6 and 12 months among the three groups (all p > 0.05), indicating convergence of stability trajectories during the later remodeling phase.

### Survival analysis

Kaplan–Meier 12-month implant survival was 98% (fully guided), 90% (pilot-drilled), and 88% (freehand) (log-rank p = 0.012). Cox regression confirmed significantly lower hazard of early failure in the fully guided group versus freehand (HR = 0.12, 95% CI 0.03–0.51, p = 0.004) and versus pilot-drilled (HR = 0.18, 95% CI 0.04–0.79, p = 0.023).

## Discussion

The study hypothesis was supported, as the fully guided technique demonstrated superior implant placement accuracy, greater stability, reduced complications, and higher patient satisfaction compared with the pilot-drilled and freehand techniques. Therefore, the study hypothesis is accepted.

Because a small number of patients in each group received two implants clinically, only one predefined index implant per patient was included in all analyses. This ensured that differences in surgical time, postoperative discomfort, stability, and satisfaction reflected the performance of the surgical technique itself rather than variability in the number of implants placed. Table 9

**Table 9:** Summary of surgical, biological, and patient-reported outcomes across techniques.

Outcome Category	Freehand	Pilot-Drilled	Fully Guided	Best-Performing Technique
**Surgery Duration**	Longest (75 min)	Moderate (60 min)	Shortest (45 min)	Fully Guided
**Intraoperative Complications**	Highest (10%)	Moderate (6.7%)	Lowest (3.3%)	Fully Guided
**Pain (VAS)**	Highest	Moderate	Lowest	Fully Guided
**Swelling**	Highest	Moderate	Lowest	Fully Guided
**Accuracy (Coronal/Apical/Angular)**	Lowest	Moderate	Highest	Fully Guided
**Implant Stability (ISQ)**	Lowest trajectory	Moderate	Highest trajectory	Fully Guided
**Marginal Bone Loss (12 months)**	Highest (2.0 ± 0.5 mm)	Moderate (1.5 ± 0.6 mm)	Lowest (0.5 ± 0.3 mm)	Fully Guided
**Aesthetic Satisfaction**	Lowest	Moderate	Highest	Fully Guided
**Overall Satisfaction**	7.2/10	8.5/10	9.2/10	Fully Guided
**Implant Survival (12 months)**	Lowest (86.7%)	Highest (100%)	High (93.3%)	Pilot-Drilled
**Biological/Mechanical Complications**	Highest	Moderate	Lowest	Fully Guided

In the field of dentistry, using dental implants to rehabilitation partial and complete edentulism is a standardized process and a common course of therapy. Its validity is demonstrated even over an extended length of time by the numerous studies in the scientific literature that report a success rate of over 95% in all dentulous clinical circumstances. [27]

The process of computer-guided implant surgery involves the use of implant planning software to virtually place the implant and choose the best option between the patient’s anatomy and the ideal implant position from both anatomical and prosthetic perspectives. This is done by uploading the patient’s three-dimensional anatomical images from the CT scan and the prosthetic information from the diagnostic wax-up scans. [27,28]

Combining the benefits of both completely guided and freehand implant placement techniques, the pilot drill guide offers a hybrid approach. Additionally, it has a number of benefits, including the ability to achieve an accuracy level comparable to that of the more exact completely guided template. [29]The proper placement of the implant is essential to its effectiveness since improper placement can change the biomechanical relationship and jeopardize the implant’s longevity. Using a stereolithographic surgical guide is one way to bring virtual planning into the operating room. It enables minimal error osteotomic drill guidance based on intended position, inclination, and depth. For an oral surgeon, measuring the frequency of these errors is crucial because it allows them to determine the level of technique accuracy and, in turn, the safety margin to be adhered to when implant planning. [30–33]

A randomized controlled trial (RCT) conducted by Younes et al compared free-handed (FH), pilot drill guided (PG) and fully guided (FG) surgery has been published. [5] In his study, partially edentulous patients were treated by either free handed (FH) surgery or by means of pilot-drill guided (PG) or fully-guided (FG) surgery using a tooth-supported guide. The results of this study indicate a significantly higher accuracy for FG and PG surgery as compared to FH surgery. These results are in line with an earlier report by Vercruyssen et al. comparing 2 systems of FG surgery to FH and template-assisted surgery in fully edentulous patients. In their study, a significant difference in accuracy was found in favour of FG surgery. [25]

The results of this study provide compelling insights into the efficacy of three different surgical techniques—freehand, pilot drilled, and fully guided implant surgery—in the management of partially edentulous patients. Each technique demonstrated unique advantages and shortcomings, which may influence clinical decision-making in practice.

### Surgical outcomes

Because a minority of patients in each group received two implants, one predefined ‘index implant’ was analyzed for each participant. This prevented confounding effects—such as increased surgical time or postoperative discomfort—associated with multi-implant procedures. Consequently, all clinical outcomes (surgical time, pain, swelling, stability, and satisfaction) accurately reflect the performance of the assigned surgical technique rather than variability in the number of implants placed.

#### Surgery duration.

One of the primary findings of this study was the variance in surgical duration among the three techniques. The fully guided approach was the most time-consuming, averaging 45 minutes. However, this may be justified by its precision and planning advantages. The fully guided technique, while faster than the pilot drilled method, yielded 100% successful placements, highlighting its effectiveness. Conversely, freehand surgery, being the least structured, resulted in longer procedure times and a slightly lower success rate (93.3%). [34]

#### Intraoperative complications.

The incidence of intraoperative complications was lowest in the fully guided group, with only one complication reported. This finding suggests that guided surgery minimizes the risk of mishaps during the procedure, likely due to enhanced visual and procedural guidance. The freehand technique had the highest number of complications (10%), which may be attributed to the increased variability in surgeon skill and experience. While freehand techniques can be effective, this underscores the importance of having well-defined protocols for complex cases. [35]

The improved clinical outcomes observed in the fully guided group may be related not only to enhanced accuracy but also to more controlled osteotomy execution and reduced soft-tissue manipulation, which are known contributors to lower postoperative morbidity.

### Postoperative recovery

#### Pain levels and swelling.

Pain was evaluated through Visual Analogue Scale (VAS) scores, revealing that patients in the freehand group experienced more significant discomfort on the first postoperative day compared to the other groups. The fully guided group had the least pain, which may relate to less traumatic surgical techniques and precision leading to reduced soft tissue disruption. Similarly, swelling measured on Day 3 reinforced these findings, with the fully guided method resulting in the least swelling. These postoperative outcomes suggest that guided techniques not only improve implant placement accuracy but also minimize trauma to surrounding tissues, leading to enhanced recovery experiences for patients. [36–39]

### Implant stability and osseointegration

The Resonance Frequency Analysis (RFA) scores indicated that fully guided implants showed superior stability at all time points assessed (3, 6, and 12 months). This aligns with current literature suggesting that precise placement is critical for optimal osseointegration, contributing to better long-term stability. The pilot drilled method showed promising results as well, maintaining a steady increase in RFA scores, indicative of good osseointegration. In contrast, the freehand group exhibited a more modest increase over time, which may reflect variability in technique and anatomical considerations that can influence results. [37]

The superior ISQ trajectory and reduced marginal bone loss in the fully guided group suggest that implants placed with higher positional precision achieve more favorable biomechanical loading after prosthetic rehabilitation, potentially reducing early bone remodeling.

### Patient satisfaction

Patient satisfaction scores were highest in the fully guided group, emphasizing the importance of experience during the immediate postoperative period. The aesthetic satisfaction ratings further corroborate these findings, potentially reflecting better positioning and alignment of implants with the fully guided approach. High satisfaction levels in the guided group could translate into better compliance with follow-up care, potentially influencing overall treatment success. [38–40]

### Long-term success rates

The long-term success rates across the three techniques highlighted the robust outcomes achievable with the pilot drilled method, with a perfect survival rate. This is particularly relevant for clinicians seeking to balance efficiency and efficacy. However, the fully guided approach closely followed, with a survival rate of 93.3%, showcasing its reliability in implant success. The freehand technique had a relatively lower survival rate (86.7%), raising concerns that may compel practitioners to reconsider its use in more complex or challenging cases. [25,41]

### Clinical implications

The choice of implant surgical technique should be tailored to individual patient needs, surgeon experience, and the specific clinical situation at hand. While fully guided techniques seem to offer a higher degree of predictability and patient comfort, they also demand significant initial investment in planning and technology. This may not be feasible for all practices; thereby, a balanced approach considering the specific circumstances of each case is vital.

The pilot drilled method provides an effective middle ground for surgeons accustomed to both traditional and guided techniques, supporting a high success rate without excessive operative time.

### Limitations and future recommendations

While the study provides valuable insights, it is not without limitations. The sample size, though adequate for preliminary findings, may be expanded in future studies to assess broader populations and diverse clinical scenarios. Long-term follow-up beyond one year is essential to ascertain the durability of these findings as implant success can be influenced by various factors over time.

Future research may also explore the cost-effectiveness of each technique, particularly as digital technologies continue to advance. Patient-reported outcomes and quality of life post- surgery should be key areas of focus to capture the full impact of these interventions.

## Conclusion

This study highlights the importance of evaluating surgical techniques in implant dentistry to enhance clinical outcomes for partially edentulous patients. Among the methods assessed— freehand, pilot-drilled, and fully guided—the fully guided technique demonstrated superior stability, patient satisfaction, and recovery. The pilot-drilled approach also delivered excellent outcomes, combining efficiency with a 100% survival rate and minimal complications. While the freehand method remains viable, it requires meticulous planning to achieve comparable results. Understanding the strengths and limitations of these techniques is crucial for advancing surgical protocols and optimizing patient care in implant dentistry.

## Supporting information

S1 Data Supporting information. (XLSX)

S1 File Trail study protocol. (DOCX)

## References

[1] Belser UC, Mericske-Stern R, Bernard JP, Taylor TD. Prosthetic management of the partially dentate patient with fixed implant restorations. Clin Oral Implants Res. 2000;11 Suppl 1:126–45. doi: 10.1034/j.1600-0501.2000.011s1126.x 11168262

[2] Buser D, Martin W, Belser UC. Optimizing esthetics for implant restorations in the anterior maxilla: anatomic and surgical considerations. Int J Oral Maxillofac Implants. 2004;19 Suppl:43–61. 15635945

[3] Ramaglia L, Toti P, Sbordone C, Guidetti F, Martuscelli R, Sbordone L. Implant angulation: 2-year retrospective analysis on the influence of dental implant angle insertion on marginal bone resorption in maxillary and mandibular osseous onlay grafts. Clin Oral Investig. 2015;19(4):769–79. doi: 10.1007/s00784-014-1275-5 24998769

[4] Tallarico M, Scrascia R, Annucci M, Meloni SM, Lumbau AI, Koshovari A, et al. Errors in Implant Positioning Due to Lack of Planning: A Clinical Case Report of New Prosthetic Materials and Solutions. Materials (Basel). 2020;13(8):1883. doi: 10.3390/ma13081883 32316361 PMC7215328

[5] Younes F, Cosyn J, De Bruyckere T, Cleymaet R, Bouckaert E, Eghbali A. A randomized controlled study on the accuracy of free-handed, pilot-drill guided and fully guided implant surgery in partially edentulous patients. J Clin Periodontol. 2018;45(6):721–32. doi: 10.1111/jcpe.12897 29608793

[6] Lambert PM, Morris HF, Ochi S. Positive effect of surgical experience with implants on second-stage implant survival. J Oral Maxillofac Surg. 1997;55(12 Suppl 5):12–8. doi: 10.1016/s0278-2391(16)31192-2 9393421

[7] Bover-Ramos F, Viña-Almunia J, Cervera-Ballester J, Peñarrocha-Diago M, García-Mira B. Accuracy of Implant Placement with Computer-Guided Surgery: A Systematic Review and Meta-Analysis Comparing Cadaver, Clinical, and In Vitro Studies. Int J Oral Maxillofac Implants. 2018;33(1):101–15. doi: 10.11607/jomi.5556 28632253

[8] Pozzi A, Polizzi G, Moy PK. Guided surgery with tooth-supported templates for single missing teeth: A critical review. Eur J Oral Implantol. 2016;9 Suppl 1:S135–53. 27314119

[9] Saurabh C, Alfarsi MA, Chaturvedi M, Pandey K, Vaddamanu SK. Immediate Implant with Simultaneous Ridge Augmentation. Journal of Dental Problems and Solutions. 2017;4:036–9. doi: 10.17352/2394-8418.000045

[10] Joda T, Derksen W, Wittneben JG, Kuehl S. Static computer-aided implant surgery (s-CAIS) analysing patient-reported outcome measures (PROMs), economics and surgical complications: A systematic review. Clin Oral Implants Res. 2018;29 Suppl 16:359–73. doi: 10.1111/clr.13136 30328203

[11] Jemt T, Johansson J. Implant treatment in the edentulous maxillae: a 15-year follow-up study on 76 consecutive patients provided with fixed prostheses. Clin Implant Dent Relat Res. 2006;8(2):61–9. doi: 10.1111/j.1708-8208.2006.00003.x 16774591

[12] Park S-J, Leesungbok R, Cui T, Lee SW, Ahn S-J. Reliability of a CAD/CAM Surgical Guide for Implant Placement: An In Vitro Comparison of Surgeons’ Experience Levels and Implant Sites. Int J Prosthodont. 2017;30(4):367–169. doi: 10.11607/ijp.5179 28697207

[13] Schulz MC, Hofmann F, Range U, Lauer G, Haim D. Pilot-drill guided vs. full-guided implant insertion in artificial mandibles-a prospective laboratory study in fifth-year dental students. Int J Implant Dent. 2019;5(1):23. doi: 10.1186/s40729-019-0176-4 31240421 PMC6593025

[14] Alevizakos V, Mitov G, Stoetzer M, von See C. A retrospective study of the accuracy of template-guided versus freehand implant placement: A nonradiologic method. Oral Surg Oral Med Oral Pathol Oral Radiol. 2019;128(3):220–6. doi: 10.1016/j.oooo.2019.01.009 31227455

[15] Marei HF, Abdel-Hady A, Al-Khalifa K, Al-Mahalawy H. Influence of surgeon experience on the accuracy of implant placement via a partially computer-guided surgical protocol. Int J Oral Maxillofac Implants. 2019;34(5):1177–83. doi: 10.11607/jomi.7480 30934035

[16] Tahmaseb A, Wu V, Wismeijer D, Coucke W, Evans C. The accuracy of static computer-aided implant surgery: A systematic review and meta-analysis. Clin Oral Implants Res. 2018;29 Suppl 16:416–35. doi: 10.1111/clr.13346 30328191

[17] Tallarico M, Esposito M, Xhanari E, Caneva M, Meloni SM. Computer-guided vs freehand placement of immediately loaded dental implants: 5-year postloading results of a randomised controlled trial. Eur J Oral Implantol. 2018;11(2):203–13. 29806667

[18] Fortin T, Isidori M, Bouchet H. Placement of posterior maxillary implants in partially edentulous patients with severe bone deficiency using CAD/CAM guidance to avoid sinus grafting: a clinical report of procedure. Int J Oral Maxillofac Implants. 2009;24(1):96–102. 19344031

[19] Pommer B, Busenlechner D, Fürhauser R, Watzek G, Mailath-Pokorny G, Haas R. Trends in techniques to avoid bone augmentation surgery: Application of short implants, narrow-diameter implants and guided surgery. J Craniomaxillofac Surg. 2016;44(10):1630–4. doi: 10.1016/j.jcms.2016.08.012 27637478

[20] Rungcharassaeng K, Caruso JM, Kan JYK, Schutyser F, Boumans T. Accuracy of computer-guided surgery: A comparison of operator experience. J Prosthet Dent. 2015;114(3):407–13. doi: 10.1016/j.prosdent.2015.04.004 26119019 PMC4486338

[21] Apparaju V, Vaddamanu SK, Vyas R, Vishwanath S, Gurumurthy V, Kanji MA. Is balloon-assisted maxillary sinus floor augmentation before dental implant safe and promising? A systematic review and meta-analysis. Niger J Clin Pract. 2020;23(3):275–83. doi: 10.4103/njcp.njcp_238_19 32134023

[22] Fürhauser R, Mailath-Pokorny G, Haas R, Busenlechner D, Watzek G, Pommer B. Esthetics of Flapless Single-Tooth Implants in the Anterior Maxilla Using Guided Surgery: Association of Three-Dimensional Accuracy and Pink Esthetic Score. Clin Implant Dent Relat Res. 2015;17 Suppl 2:e427–33. doi: 10.1111/cid.12264 25346154

[23] Younes F, Eghbali A, De Bruyckere T, Cleymaet R, Cosyn J. A randomized controlled trial on the efficiency of free-handed, pilot-drill guided and fully guided implant surgery in partially edentulous patients. Clin Oral Implants Res. 2019;30(2):131–8. doi: 10.1111/clr.13399 30578650

[24] Talluri S, Vaddamanu SK, Apparaju V, Vyas R, Ahuja S, Kanji MA. Evaluating cortico-cancellous ratio using virtual implant planning and its relation with immediate and long-term stability of a dental implant- A CBCT-assisted prospective observational clinical study. Niger J Clin Pract. 2019;22(7):982–7. doi: 10.4103/njcp.njcp_22_19 31293265

[25] Vercruyssen M, Cox C, Coucke W, Naert I, Jacobs R, Quirynen M. A randomized clinical trial comparing guided implant surgery (bone- or mucosa-supported) with mental navigation or the use of a pilot-drill template. J Clin Periodontol. 2014;41(7):717–23. doi: 10.1111/jcpe.12231 24460748

[26] Hopewell S, Chan AW, Collins GS, Hróbjartsson A, Moher D, Schulz KF. CONSORT 2025 statement: updated guideline for reporting randomised trials. BMJ. 2025. doi: e081123 10.1136/bmj-2024-081123 PMC11995449 40228833

[27] Schneider D, Sancho-Puchades M, Schober F, Thoma D, Hämmerle C, Jung R. A Randomized Controlled Clinical Trial Comparing Conventional and Computer-Assisted Implant Planning and Placement in Partially Edentulous Patients. Part 3: Time and Cost Analyses. Int J Periodontics Restorative Dent. 2019;39(3):e71–82. doi: 10.11607/prd.4146 30986285

[28] Behneke A, Burwinkel M, Behneke N. Factors influencing transfer accuracy of cone beam. 2023. CT-derived template-based implant placement. Clin Oral Implants Res. 2012;23:416–23. doi: 10.1111/j.1600-0501.2011.02337.x 22092586

[29] Fang Y, An X, Jeong S-M, Choi B-H. Accuracy of computer-guided implant placement in anterior regions. The Journal of Prosthetic Dentistry. 2019;121(5):836–42. doi: 10.1016/j.prosdent.2018.07.015 30598309

[30] Van Assche N, Quirynen M. Tolerance within a surgical guide. Clin Oral Implants Res. 2010;21(4):455–8. doi: 10.1111/j.1600-0501.2009.01836.x 20074247

[31] Vercruyssen M, van de Wiele G, Teughels W, Naert I, Jacobs R, Quirynen M. Implant- and patient-centred outcomes of guided surgery, a 1-year follow-up: An RCT comparing guided surgery with conventional implant placement. J Clin Periodontol. 2014;41(12):1154–60. doi: 10.1111/jcpe.12305 25197015

[32] Albrektsson T. A multicenter report on osseointegrated oral implants. J Prosthet Dent. 1988;60(1):75–84. doi: 10.1016/0022-3913(88)90355-1 3042986

[33] Lekholm U, Gunne J, Henry P, Higuchi K, Lindén U, Bergström C, et al. Survival of the Brånemark implant in partially edentulous jaws: a 10-year prospective multicenter study. Int J Oral Maxillofac Implants. 1999;14(5):639–45. 10531735

[34] Ekelund JA, Lindquist LW, Carlsson GE, Jemt T. Implant treatment in the edentulous mandible: a prospective study on Brånemark system implants over more than 20 years. Int J. 2023;34(1):1–10. 14714838

[35] Pjetursson BE, Sailer I, Zwahlen M, Hämmerle CHF. A systematic review of the survival and complication rates of all-ceramic and metal-ceramic reconstructions after an observation. Clinical Oral Implants Research. 2007;73–85. doi: 10.1111/j.1600-0501.2007.01467.x 17594372

[36] De Santis D, Graziani P, Castellani R, Zanotti G, Gelpi F, Marconcini S, et al. A New Radiologic Protocol and a New Occlusal Radiographic Index for Computer-Guided Implant Surgery. J Craniofac Surg. 2016;27(5):e506–10. doi: 10.1097/SCS.0000000000002490 27391524

[37] Hobkirk JA, Havthoulas TK. The influence of mandibular deformation, implant numbers, and loading position on detected forces in abutments supporting fixed implant superstructures. J Prosthet Dent. 1998;80(2):169–74. doi: 10.1016/s0022-3913(98)70106-4 9710818

[38] Stanford CM. Biomechanical and functional behavior of implants. Adv Dent Res. 1999;13:88–92. doi: 10.1177/08959374990130012101 11276753

[39] Kopp KC, Koslow AH, Abdo OS. Predictable implant placement with a diagnostic/surgical template and advanced radiographic imaging. J Prosthet Dent. 2003;89(6):611–5. doi: 10.1016/s0022-3913(03)00198-7 12815357

[40] Marlière DAA, Demètrio MS, Picinini LS, Oliveira RGD, Netto HDDMC. Accuracy of computer-guided surgery for dental implant placement in fully edentulous patients: A systematic review. Eur J Dent. 2018;12(1):153–60. doi: 10.4103/ejd.ejd_249_17 29657542 PMC5883470

[41] Vercruyssen M, Coucke W, Naert I, Jacobs R, Teughels W, Quirynen M. Depth and lateral deviations in guided implant surgery: an RCT comparing guided surgery with mental navigation or the use of a pilot-drill template. Clin Oral Implants Res. 2015;26(11):1315–20. doi: 10.1111/clr.12460 25179585

