# Supplementary material for: The efficacy of freehand, pilot drilled and fully guided implant surgery in partially edentulous patients: A randomized control trial
Source: PLoS One. 2026 Jan 27;21(1):e0341894. doi: 10.1371/journal.pone.0341894 (PMC12843514; doi:10.1371/journal.pone.0341894)
Supplement: S1 File — (DOCX) [file pone.0341894.s002.docx]

**Pre-Request Proposal for Ethical Approval**

**Title**: Efficacy of Freehand, Pilot-Drilled, and Fully Guided Implant Surgery in Partially Edentulous Patients: A Randomized Controlled Trial

**Principal Investigator**: Sunil Kumar Vaddamanu, Department of Allied Dental Health Sciences, College of Applied Medical Sciences, King Khalid University, Abha, Saudi Arabia
**Co-Investigators**: Abdulkhaliq Ali F Alshadidi, Lujain Ibrahim N Aldosari, Abdullah Hasan A Alshehri, Rayan Ibrahim H Binduhayym, Rajamanoj Kondaveeti, Vishwanath Gurumurthy

**Institution**: King Khalid University, Abha, Saudi Arabia

**Submission Date**: January 5, 2025

**Proposed Study Period**: January 6, 2025 – August 3, 2025

**ClinicalTrials.gov Identifier**: NCT06764784

**1. Background and Rationale**

Partial edentulism significantly impacts oral function, aesthetics, and quality of life. Dental implants are a standard treatment, but their success depends on the surgical technique used—freehand, pilot-drilled, or fully guided. These techniques vary in accuracy, surgical time, complications, and patient satisfaction. Limited comparative data exist on their efficacy in partially edentulous patients, particularly using standardized protocols. This randomized controlled trial (RCT) aims to compare these techniques to guide clinical practice, addressing a critical gap in evidence-based dentistry.

The study is necessary to:

- Evaluate the precision and safety of implant placement techniques.
- Assess patient-centered outcomes, such as satisfaction and postoperative recovery.
- Inform evidence-based decision-making in implant dentistry.

**2. Study Objectives**

**Primary Objective**: To compare the efficacy of freehand, pilot-drilled, and fully guided implant surgery in partially edentulous patients with respect to surgical outcomes (duration, complications), postoperative recovery (pain, swelling), implant stability, osseointegration, and patient satisfaction.

**Secondary Objectives**: To evaluate long-term implant success rates and summarize the advantages and limitations of each technique to optimize clinical protocols.

**3. Study Design**

This is a single-center, parallel-arm RCT involving 90 partially edentulous patients, randomly assigned to three groups (n=30 each):

1. **Freehand Implant Surgery (FHIS)**: Implants placed using clinical judgment and visual landmarks.
2. **Pilot-Drilled Implant Surgery (PDIS)**: Initial osteotomy guided by a pilot drill, verified by fluoroscopy or intraoral guides.
3. **Fully Guided Implant Surgery (FGIS)**: Implants placed using customized surgical guides based on preoperative CBCT and planning software.

**Key Outcomes**:

- Surgical duration (minutes).
- Implant placement accuracy (via postoperative CBCT).
- Postoperative complications (within 30 days).
- Early implant failure rates (within 6 months).
- Patient satisfaction (via validated 10-point questionnaire at 6 months).
- Implant stability (via Resonance Frequency Analysis at 3, 6, and 12 months).
- Pain and swelling (via Visual Analogue Scale on Days 1 and 3).

**Study Duration**: January 6, 2025 (recruitment start) to August 3, 2025 (final follow-up).
**Sample Size**: 90 patients (30 per group), calculated for 80% power and alpha of 0.05, assuming a medium effect size (Cohen’s d = 0.5) for implant placement accuracy, with a 10% dropout rate.

**4. Participant Recruitment**

**Inclusion Criteria**:

- Adults aged ≥18 years.
- Partial edentulism (missing one or more teeth in an arch with remaining natural teeth).
- Sufficient bone volume and quality for implant placement (confirmed by CBCT).
- No systemic contraindications for surgery.

**Exclusion Criteria**:

- Uncontrolled systemic diseases (e.g., uncontrolled diabetes).
- Smokers or patients with a history of non-compliance with postoperative care.
- Patients requiring bone grafting or sinus lifts.

**Recruitment Process**: Patients will be recruited from dental clinics at King Khalid University. Eligible patients will receive detailed study information and provide written informed consent. Recruitment will occur between January 6 and February 3, 2025.

**5. Ethical Considerations**

This study adheres to the Declaration of Helsinki and Good Clinical Practice guidelines. Key ethical considerations include:

**Informed Consent**:

- All participants will provide written informed consent after receiving comprehensive information about the study’s purpose, procedures, risks, benefits, and their right to withdraw at any time without penalty.
- Consent forms will be available in Arabic and English, ensuring accessibility.

**Risks and Benefits**:

- **Risks**: Potential risks include surgical complications (e.g., infection, nerve injury), pain, swelling, and implant failure, which are inherent to dental implant procedures. These will be minimized through standardized protocols, experienced surgeons, and rigorous postoperative monitoring.
- **Benefits**: Participants will receive high-quality implant treatment at no cost, potentially improving oral function and aesthetics. The study will contribute to evidence-based implant dentistry, benefiting future patients.

**Confidentiality**:

- Patient data will be anonymized using unique identifiers. Data will be stored in a secure, password-protected database accessible only to authorized personnel.
- Only de-identified data will be used for analysis and publication.

**Adverse Event Management**:

- Adverse events (e.g., complications, implant failure) will be monitored and reported to the ethics committee within 24 hours.
- Participants experiencing adverse events will receive immediate clinical care per standard protocols.

**Voluntary Participation**:

- Participation is voluntary, and patients can withdraw at any time without affecting their access to standard care.

**6. Methodology**

**Randomization**: Computer-generated randomization (1:1:1 ratio), stratified by age and gender, will assign participants to FHIS, PDIS, or FGIS groups.

**Blinding**: Surgeons and participants cannot be blinded due to the nature of the interventions. Outcome assessors (radiologists, statisticians) will be blinded to group allocation to reduce bias.
**Preoperative Assessment**:

- Comprehensive dental and medical history.
- Clinical examination and CBCT for bone assessment.
- Impressions for study models to plan implant positions.

**Interventions**:

- **FHIS**: Implants placed using clinical judgment, with minimal surgical guides.
- **PDIS**: Pilot drill used for initial osteotomy, followed by sequential drilling.
- **FGIS**: Customized surgical guides based on CBCT and planning software ensure precise implant placement.

**Data Collection**:

- Baseline: Demographics, medical history, CBCT findings.
- Intraoperative: Surgical duration, complications.
- Postoperative: Pain, swelling, complications, RFA scores, and patient satisfaction at specified intervals.
- Data will be recorded in standardized case report forms and stored securely.

**Statistical Analysis**:

- Descriptive statistics: Mean, SD for continuous variables; frequencies for categorical variables.
- Normality testing: Shapiro-Wilk test.
- Comparative analysis: One-way ANOVA with Tukey post hoc test for group differences.
- Subgroup analysis: Canine vs. molar implants.
- Software: SPSS (Version 23).
- Significance level: $p < 0.05$.
- Analysis will follow an intention-to-treat approach.

**7. Data Management and Monitoring**

- Data will be stored in a secure electronic database with regular backups.
- An independent data monitoring committee will oversee trial safety and protocol adherence.
- Regular audits will ensure data accuracy and compliance with ethical standards.

**8. Justification for Ethical Approval**

This study addresses a critical gap in comparative data on implant surgical techniques, with potential to improve clinical outcomes and patient satisfaction. The trial poses minimal additional risk beyond standard implant procedures, as all techniques are established in clinical practice. Ethical safeguards include:

- Rigorous informed consent process.
- Minimization of risks through standardized protocols and experienced surgeons.
- Robust data protection measures.
- Immediate reporting and management of adverse events.
- Adherence to international ethical standards.

The study’s findings will contribute to evidence-based dentistry, potentially improving treatment protocols for partially edentulous patients globally.

**9. Funding**

Funded by the Deanship of Scientific Research at King Khalid University (Grant Number RGP1/3/46).

**10. Declarations**

- **Ethics Compliance**: The study will comply with the Declaration of Helsinki.
- **Competing Interests**: The investigators declare no competing interests.
- **Data Availability**: All relevant data will be included in the published manuscript, ensuring transparency.

**11. Supporting Documents**

- Informed consent form (in Arabic and English).
- Participant information sheet.
- Case report forms.
- Draft questionnaire for patient satisfaction.
- CBCT imaging protocol.

**12. Contact Information**

**Principal Investigator**: Sunil Kumar Vaddamanu
Email: [snu@kku.edu.sa](mailto:snu@kku.edu.sa)
Phone: +966-595220377
**Institution**: King Khalid University, Abha, Saudi Arabia

**CBCT Imaging Protocol**

**Title**: Cone Beam Computed Tomography (CBCT) Imaging Protocol for the Randomized Controlled Trial on Efficacy of Freehand, Pilot-Drilled, and Fully Guided Implant Surgery in Partially Edentulous Patients

**Study Reference**: ClinicalTrials.gov Identifier NCT06764784

**Institution**: King Khalid University, Abha, Saudi Arabia

**Date**: January 5, 2025

**1. Purpose**

The CBCT imaging protocol aims to:

- Provide accurate preoperative assessment of bone anatomy and volume for implant planning.
- Verify postoperative implant placement accuracy by comparing planned and actual implant positions.
- Ensure consistent imaging parameters to minimize variability and radiation exposure while maintaining diagnostic quality.

**2. Scope**

This protocol applies to all 90 participants enrolled in the RCT, covering preoperative and postoperative CBCT scans for the freehand (FHIS), pilot-drilled (PDIS), and fully guided (FGIS) implant surgery groups.

**3. Equipment**

- **CBCT Scanner**: Carestream CS 9600 or equivalent, calibrated to manufacturer specifications.
- **Software**: Carestream 3D Imaging Software (or compatible software) for image acquisition, processing, and analysis.
- **Calibration**: The CBCT unit will be calibrated weekly to ensure consistent image quality and radiation output, following manufacturer guidelines and ALARA (As Low As Reasonably Achievable) principles.

**4. Imaging Parameters**

To ensure reproducibility and diagnostic accuracy, the following standardized parameters will be used:

- **Field of View (FOV)**:
  - Small FOV (5x5 cm or 8x8 cm) for single-tooth or small-segment edentulous areas.
  - Medium FOV (10x10 cm) for larger edentulous spans or multiple implants, ensuring inclusion of adjacent teeth and anatomical landmarks (e.g., inferior alveolar nerve, maxillary sinus).
- **Voxel Size**: 0.2 mm for high-resolution imaging to assess fine bone details and implant positioning.
- **kVp**: 90 kVp (range: 80–100 kVp, adjusted based on patient size to optimize image quality).
- **mA**: 5–10 mA, adjusted to minimize radiation dose while ensuring diagnostic clarity.
- **Exposure Time**: 10–15 seconds, depending on the FOV and patient cooperation.
- **Rotation**: 360° for comprehensive 3D reconstruction.
- **Image Format**: DICOM (Digital Imaging and Communications in Medicine) for compatibility with implant planning software.

**5. Preoperative CBCT Procedure**

**Purpose**: To assess bone volume, density, and anatomical structures for implant planning. **Timing**: Performed within 2 weeks before surgery.

**Procedure**:

1. **Patient Preparation**:
   - Remove all metallic objects (e.g., jewelry, removable dentures) to avoid artifacts.
   - Provide a lead apron with a thyroid collar to minimize radiation exposure to non-target areas.
   - Position the patient upright in the CBCT scanner, with the head stabilized using a chin rest and head straps to prevent movement.
   - Instruct the patient to remain still and avoid swallowing during the scan.
2. **Scan Acquisition**:
   - Center the FOV on the edentulous area, including adjacent teeth and critical anatomical structures (e.g., inferior alveolar nerve, mental foramen, maxillary sinus).
   - Use a scout view to confirm correct positioning before the full scan.
   - Perform the scan using the parameters specified above.
3. **Image Processing**:
   - Reconstruct images in axial, coronal, and sagittal planes.
   - Export DICOM files to implant planning software (e.g., NobelClinician or Blue Sky Plan) for virtual implant placement and surgical guide fabrication (for FGIS group).
4. **Quality Check**:
   - A radiologist will review images for clarity, absence of artifacts, and inclusion of all relevant anatomical structures.
   - Repeat scans if motion artifacts or positioning errors are detected (anticipated in <5% of cases).

**6. Postoperative CBCT Procedure**

**Purpose**: To evaluate implant placement accuracy by comparing actual implant positions to preoperative plans. **Timing**: Performed within 1 week post-surgery.

**Procedure**:

1. **Patient Preparation**: Same as preoperative procedure.
2. **Scan Acquisition**:
   - Use the same FOV and parameters as the preoperative scan to ensure comparability.
   - Focus on the implant site, including surrounding bone and adjacent teeth.
3. **Image Analysis**:
   - Measure deviations in implant position (mesiodistal, buccolingual, and angular) and depth (mm) using superimposition of preoperative and postoperative images.
   - Assess osseointegration indicators (e.g., bone-implant contact) and detect complications (e.g., bone perforation).
   - Analysis will be performed by a blinded radiologist to minimize bias.

**7. Radiation Safety**

- **ALARA Principle**: Radiation doses will be minimized by using the smallest FOV necessary and optimizing kVp/mA settings.
- **Dose Estimation**: Typical effective dose for small/medium FOV scans is 50–150 µSv, well below the threshold for significant risk.
- **Patient Information**: Patients will be informed about the low radiation risk and necessity of CBCT for treatment planning and evaluation.
- **Staff Training**: Only certified radiographers will operate the CBCT scanner, following radiation safety protocols.

**8. Data Management**

- **Storage**: DICOM files will be stored in a secure, password-protected database with unique patient identifiers to ensure confidentiality.
- **Access**: Only authorized study personnel (radiologists, investigators) will access imaging data.
- **Analysis**: Images will be analyzed using validated software to quantify implant placement accuracy and bone characteristics.
- **Retention**: Imaging data will be retained for 5 years post-study, per institutional guidelines, and then securely deleted.

**9. Quality Assurance**

- **Calibration**: Weekly calibration of the CBCT scanner to maintain image quality and radiation accuracy.
- **Standardization**: All scans will follow the same protocol to ensure consistency across participants.
- **Blinding**: Radiologists analyzing postoperative images will be blinded to the surgical technique to reduce bias.
- **Audit**: An independent reviewer will audit 10% of scans to verify protocol adherence and image quality.

**10. Ethical Considerations**

- **Informed Consent**: Patients will be informed about the purpose, benefits, and low radiation risks of CBCT imaging as part of the study consent process.
- **Minimizing Exposure**: Only necessary scans (one preoperative, one postoperative) will be performed.
- **Confidentiality**: All imaging data will be anonymized and stored securely, in compliance with the Declaration of Helsinki and institutional ethics guidelines (ECM#2024-3195).

**11. Personnel**

- **Radiologist**: Responsible for image acquisition, quality control, and analysis.
- **Radiographer**: Trained to operate the CBCT scanner and ensure patient positioning.
- **Investigators**: Oversee integration of CBCT data into surgical planning and outcome assessment.

**12. Documentation**

- CBCT scan parameters and findings will be recorded in standardized case report forms.
- A log of all scans, including date, time, and any adverse events (e.g., need for repeat scans), will be maintained.
